# Diversity of active root-associated methanotrophs of three emergent plants in a eutrophic wetland in northern China

**DOI:** 10.1186/s13568-020-00984-x

**Published:** 2020-03-14

**Authors:** Jing Cui, Ji Zhao, Zheng Wang, Weiwei Cao, Shaohua Zhang, Jumei Liu, Zhihua Bao

**Affiliations:** 1 School of Life Sciences, Inner Mongolia University, Hohhot, 010070 China; 2 Ministry of Education Key Laboratory of Ecology and Resource Use of the Mongolian Plateau & Inner Mongolia Key Laboratory of Grassland Ecology, School of Ecology and Environment, Inner Mongolia University, 235 West University Street, Hohhot, 010021 China; 3 Inner Mongolia Key Laboratory of Environmental Pollution Control & Waste Resource Reuse, Inner Mongolia University, Hohhot, 010021 China; 4 College of Chemistry and Environmental Engineering, Chongqing Key Laboratory of Environmental Materials & Remediation Technologies, Chongqing University of Arts and Sciences, Chongqing, 402160 China

**Keywords:** Active methanotrophs, High-throughput sequencing, Emergent plant, Microhabitat

## Abstract

Root-associated aerobic methanotrophs play an important role in regulating methane emissions from the wetlands. However, the influences of the plant genotype on root-associated methanotrophic structures, especially on active flora, remain poorly understood. Transcription of the *pmo*A gene, encoding particulate methane monooxygenase in methanotrophs, was analyzed by reverse transcription PCR (RT-PCR) of mRNA isolated from root samples of three emergent macrophytes, including *Phragmites australis*, *Typha angustifolia*, and *Schoenoplectus triqueter* (syn. *Scirpus triqueter* L.) from a eutrophic wetland. High-throughput sequencing of *pmo*A based on DNA and cDNA was used to analyze the methanotrophic community. Sequencing of cDNA *pmo*A amplicons confirmed that the structure of active methanotrophic was not always consistent with DNA. A type I methanotroph, *Methylomonas*, was the most active group in *P. australis*, whereas *Methylocystis,* a type II methanotroph, was the dominant group in *S. triqueter*. In *T. angustifolia*, these two types of methanotroph existed in similar proportions. However, at the DNA level, *Methylomonas* was predominant in the roots of all three plants. In addition, vegetation type could have a profound impact on root-associated methanotrophic community at both DNA and cDNA levels. These results indicate that members of the genera *Methylomonas* (type I) and *Methylocystis* (type II) can significantly contribute to aerobic methane oxidation in a eutrophic wetland.

## Key points

1. Root-associated *Methylomonas* was predominant in three macrophytes using DNA approach.
2. Active *Methylocystis* was dominant in genera *Typha* and *Schoenoplectus* but not in *Phragmites.*
3. Plant species impact on methanotrophic communities in both DNA and cDNA levels.

## Introduction

Wetlands can both produce and absorb greenhouse gases, which is a major component in the global climate change. Being the largest natural wetland at the same latitude of the earth, Wuliangsuhai (WLSH) is a typical eutrophication wetland in northern China (Wu et al. 2017) and plays an important role in the earth’s ecosystem, such as maintaining water resources, regulating drought climate, and providing high biodiversity, etc. (Liu et al. 2020; Yu et al. 2004). Methane, a greenhouse gas, accounts for 20–30% of the contribution of greenhouse gases to global warming (Conrad 2009). In nature, methane is normally produced by methanogens in anaerobic zone of soil (Serrano-Silva et al. 2014), but is not directly released into the atmosphere. About 90% is consumed by methanotrophic bacteria when passing through the aerobic soil layer. As a biofilter, methanotrophs became a powerful biological weapon to combat global climate change (Hornibrook et al. 2009). Aerobic methanotrophs, with the help of a series of enzymes, can eventually convert particulate methane into carbon dioxide, thus effectively reducing the greenhouse effect of methane. Methane monooxygenase (pMMO) enzyme plays an important role in this process. As a key gene encoding the β-subunit of pMMO, *pmo*A was found in almost all known aerobic methanotrophs. So far, the diversity of methanotrophs is typically assessed by the detection of the *pmo*A gene (Brablcova et al. 2015; McDonald et al. 2008; Semrau et al. 2010).

Eutrophication is a serious ecology problem in major aquatic ecosystems around the world, and aquatic macrophytes play critical roles in improving water quality (Dhote and Dixit 2009). *Phragmites* spp., *Typha* spp., and *Schoenoplectus* spp. are three common emergent types of macrophyte vegetation that are present worldwide (Vymazal 2013), and they mediate CH_4_ emissions from wetlands to the atmosphere (Grunfeld and Brix 1999). A well-developed aeration tissue inside the emergent plants can mediate the release of methane from the sediments into the atmosphere through the plants. In this process, the methanotrophs in the plant roots will have a decisive influence on the final methane gas emissions. As was observed in constructed wetlands, the existence of plants play an important role in regulating the production, consumption and transportation of CH_4_ (Sun et al. 2013). CH_4_ flux is usually measured under the condition of constructed wetland, which is disturbed by human factors (Zhang et al. 2018). For CH_4_ emissions, published results report lower (Bateganya et al. 2015; Maltais-Landry et al. 2009) or higher (Wang et al. 2013) in planted compared to unplanted constructed wetlands. However, the effect of different plant species on CH_4_ fluxes remains controversial (Chen et al. 2019). Between 18 and 90% of the produced CH_4_ in the root zone of emergent macrophytes in wetlands is consumed by aerobic methanotrophs (Grunfeld and Brix 1999; Laanbroek 2010). So far, many studies have focused on microorganisms in their rhizosphere sediment, including methylotroph- and heterotroph-mediated processes of carbon and other element cycles (Borruso et al. 2017). Through a 16S rRNA gene Illumina MiSeq sequencing, Pietrangelo et al. (2018) reported the bacterial community structure on the root surface of *P. australis* was indeed different from that of *T. latifolia*. In addition, Fausser et al. (2012) have suggested that methylotrophic bacteria live in the root zones of *P. australis* and *T. latifolia*. However, the community structure of root-associated methanotrophs and the relationship between the root-associated methanotrophs (functional bacteria) and plant species remain poorly understood.

As more and more molecular biology techniques are applied to the environment, reverse transcription polymerase chain reaction (RT-PCR) is another useful tool to identify active methanotrophs in the environment (Burgmann et al. 2001; Chen et al. 2007; Esson et al. 2016; Griffiths et al. 2000). Different from the measurement of methane flux, studying the transcriptional activity of functional gene *pmo*A can help us understand the activities of aerobic methanotrophs directly. At the same time, transcriptional analysis from natural wetland samples without laboratory culture can more accurately reflect the community characteristics of aerobic methanotrophs in their natural state.

WLSH Lake is located near the city of Bayannur in the Inner Mongolia Autonomous Region in China. This lake is the largest freshwater lake in the Yellow River watershed. Recently, the lake has become eutrophic after having received industrial wastewater with high nitrogen and phosphorus content (Wu et al. 2017). *P. australis* (common reed), *T. angustifolia* (narrow leaf cattail), and *S. triqueter* (bulrush) are the dominant macrophytes of WLSH (Duan et al. 2005).

Using the RT-PCR and MiSeq sequencing technique, we studied the structure of methanotrophic communities in the roots of three typical emergent plants (*P. australis*, *T. angustifolia*, and *S. triqueter*) in WLSH wetland, Inner Mongolia of China. This study was conducted to determine (1) whether any trends in active aerobic methanotrophs can be identified based on *pmo*A sequence analysis of cDNA, and (2) whether plant species influence the structure of methanotrophs. The results will be valuable for discussions and decisions related to the emission of greenhouse gas and the restoration of ecosystems by plants in WLSH wetland.

## Materials and methods

### Sampling sites and plant materials

**Fig. 1:**
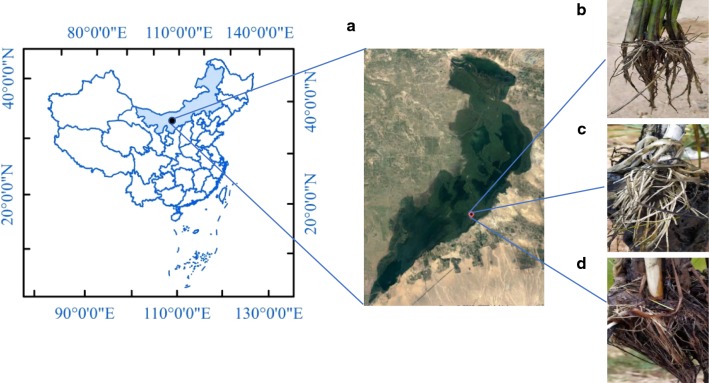
Sampling site (**a**) of *P. australis* (**b**), *T. angustifolia* (**c**) and *S. triqueter* (**d**) in the natural wetland of Wuliangsuhai

Three plants of each *P. australis*, *T. angustifolia* and *S. triqueter* were collected from WLSH wetland (N 40°52′36″, E 108°51′16″) in 15 July 2017 (Fig. 1). The physical and chemical properties of the sampling site are shown in the attached Table (Additional file 1: Table S1). The roots of the three plants were collected from the wetland located in the naturalistic area of WLSH, then washed carefully with sterile water until all the soil was rinsed off. Some of the roots were carefully picked with sterilized forceps and divided into two equal parts which placed into 50 ml Falcon tubes (Bao et al. 2014a). One was quickly transferred to dry ice for DNA extraction, and the other was placed in a liquid nitrogen tank to extract RNA. We made three parallel lines for each sample, and quickly brought the tubes back to the lab. All samples were stored at − 80 °C prior to molecular analysis.

### DNA and RNA extraction and cDNA synthesis

The tissues of the roots were ground to powder in liquid nitrogen, and stored at − 80 °C until molecular analysis (Bao et al. 2014a). Genomic DNA was extracted from 0.5 g root by using the Fast DNA SPIN for soil kit (MP Biomedicals, Solon, OH), while RNA was by RNAprep pure Plant Kit (Tiangen Biotech Co., Beijing), and both extraction processes were done according to the manufacturer’s instructions.

Though DNase was added in the process of RNA extraction, host DNA pollution would have a great impact on the test results. The following methods (Zhao et al. 2018) were used to detect DNA contamination: primer set 27F/1492R for 16S rRNA (Martin-Laurent et al. 2001) gene was used for PCR amplification with the template of the extracted total RNA. The PCR products were analyzed as negative results by agarose gel electrophoresis and NanoVueTM Plus (GE, USA) to ensure that there was no microbial DNA in the total RNA. gDNA eraser reverse transcription kit (TaKaRa, Japan) was used to synthesize cDNA according to the manufacturer’s instructions. The first step of gDNA eraser reverse transcription kit was genomic DNA elimination reaction which can make sure no DNA left. The reagents, gDNA eraser, used in the reaction have a strong decomposition effect on DNA. The second step was reverse-transcription reaction. The processes were also followed the protocol, and the primers were RT primer mix (olige dT and random 6 mers). All the DNA and cDNA samples were stored at − 80 °C until use.

### High-throughput sequencing of *pmo*A genes based on both DNA and cDNA

The *pmo*A gene in DNA and cDNA samples from three plants root were sequenced using the Illumina MiSeq platform. The barcode primer pair A189f/mb661r (Costello and Lidstrom 1999) and reagent kit (RR902A, Premix ExTaq™, Takara Bio Inc., Japan) were used for PCR amplification. A raw sequence file was processed by using the mothur software (version 1.33.3) for quality control and sample splitting (Schloss et al. 2009). The reads were processed using the online version of FunGene Pipeline (Fish et al. 2013), then high-quality *pmo*A sequences of each sample were classified as known *pmo*A groups or lineages as described (Luke and Frenzel 2011). The nucleotide sequences of *pmo*A were clustered into species-level operational taxonomic units (OTUs) using the FunGene Pipeline with a distance cutoff of 0.09 (Heyer et al. 2002).

### Statistical analysis

The phylogenetic tree of the OTUs was drawn with the neighborhood joining method using MEGA5.2 (Tamura et al. 2011). The display and annotation of the tree were done with ITOL(http://itol.embl.de/). The representative sequence of OTUs were blasted in NCBI. Statistical analysis and data visualization were carried out in R (version3.6.1). Mothur software (version1.33.3) was used for calculating alpha diversity indices which were then analyzed using ANOVA (SPSS v16.0). Principal coordinates analysis (PCoA) and analysis of similarities (ANOSIM) were done by the package vegan. The package ggplot2 was used to draw the plot of PCoA. Heatmap were also plotted in R with the package pheatmap.

### Nucleotide sequence accession numbers

All data from MiSeq sequencing of the *pmo*A have been deposited in the NCBI under the accession numbers: SRR10584604-SRR10584613.

## Results

### Comparison of diversity and community of methanotrophs between DNA and cDNA amplicons

High throughput sequencing of *pmo*A gene was performed on root DNA and cDNA of three plants, and 109,563 high quality reads (DNA 59,742; cDNA 53,344) were obtained. The alpha diversity is shown in Table 1, Fig. 5a and Additional file 1: Fig. S1. The OTUs richness was evaluated through the Chao1 index whereas the OTUs evenness was evaluated through Shannon. *P. australis* had the most OTUs richness, while *T. angustifolia* had most diversity. In both alpha diversity measures, the methanotrophic diversity in roots of the three plants was significantly different from each other (p < 0.05). In DNA, bacterial diversity of *P. australis* was significantly higher than that of *T. angustifolia* and *S. triqueter* and it was lowest in *T. angustifolia*; while in cDNA, bacterial diversity of *T. angustifolia* was heights and it was significantly higher than that of *P. australis* and *S. triqueter* (Table 1, Fig. 5a).

**Table 1:** The diversity index of high-throughput sequencing based on *pm*oA of three plant roots

	DNA			cDNA		
	PAR	TAR	STR	PAR	TAR	STR
Sequences	5873 ± 177	6907 ± 495	7134 ± 105	6126 ± 289	5783 ± 278	5872 ± 355
ChaoΙ	17.50 ± 2.02	17.17 ± 3.56	19.17 ± 0.73	36.33 ± 2.73^a^	22.33 ± 2.03^ab^	17 ± 2.08^c^
Simpson	0.37 ± 0.010^a^	0.57 ± 0.012^b^	0.63 ± 0.004^c^	0.22 ± 0.008^a^	0.49 ± 0.012^b^	0.17 ± 0.003^c^
Shannon	1.31 ± 0.036^a^	0.87 ± 0.018^b^	0.94 ± 0.009^c^	1.90 ± 0.049^a^	2.11 ± 0.030^b^	1.23 ± 0.015^c^

Values are means ± standard errors (n = 3). Different letter (a, b or c) between different plant species within DNA or cDNA amplicon are significantly different (p < 0.05). PAR, *P. australis* root; TAR, *T. angustifolia* root; STR, *S. triqueter* root

**Fig. 2:**
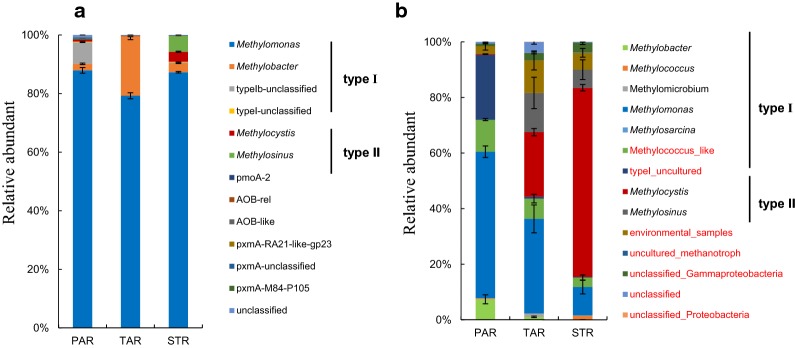
Relative abundant of aerobic methanotroph community structure in different emergent plants at the genus level based on DNA (**a**) and cDNA (**b**) analysis of *pmo*A (PAR, *P. australis* root; TAR, *T. angustifolia* root; STR, *S. triqueter* root)

**Fig. 3:**
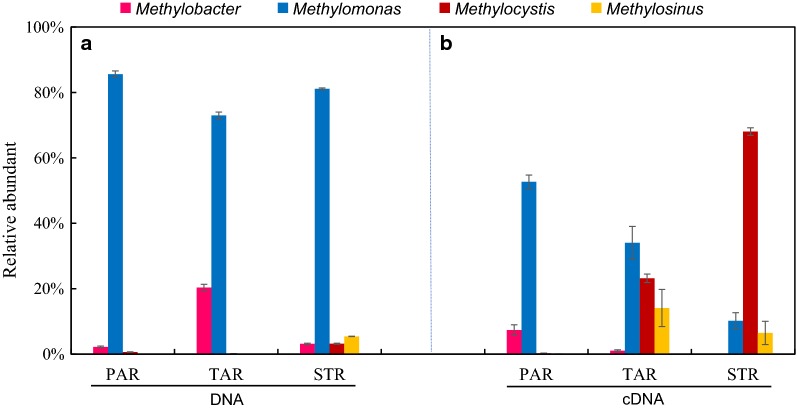
Relative abundant of four main genera in both DNA(a) and cDNA(b)

Figure 2 showed the community structure at the genus level between DNA and cDNA. For DNA, more than 90% aerobic methanotrophic bacteria belonged to type I methanotrophs affiliated with *Methylomonas* in three plants root (Fig. 2a). The relative abundance of type II in all the samples was very low (0.2–8.9%). *Methylocystis* (8.8%) in *S. triqueter* was much more than the other two plants. Unlike DNA, *Methylomonas* was still dominant in *P. australis*, and *Methylocystis* had the highest abundance in the *S. triqueter* in cDNA (Fig. 2b). The relative abundance of four main genera in both DNA and cDNA were shown in Fig. 3. As *Methylomonas* of all three plants root had the highest abundance in DNA (79.3–87.9%), it all significantly decreased to some extent in cDNA (10.2–52.7%); however, *Methylocystis* and *Methylosinus* had both significantly increased in *S. triqueter* (68%) and *T. angustifolia* (23.2%).

PCoA (Fig. 5b) were used to analyze beta-diversity of root-associated methanotrophs based on DNA and cDNA analysis. It verified that samples from the same emergent plants tended to group together, while samples from different plant species were located far apart. The difference between groups was much larger than that within groups (anosim, p < 0.01). The result clearly showed that plant species had affect the community structure of methanotrophs.

### Phylogeny of active methanotrophs associated with emergent plant roots

**Fig. 4:**
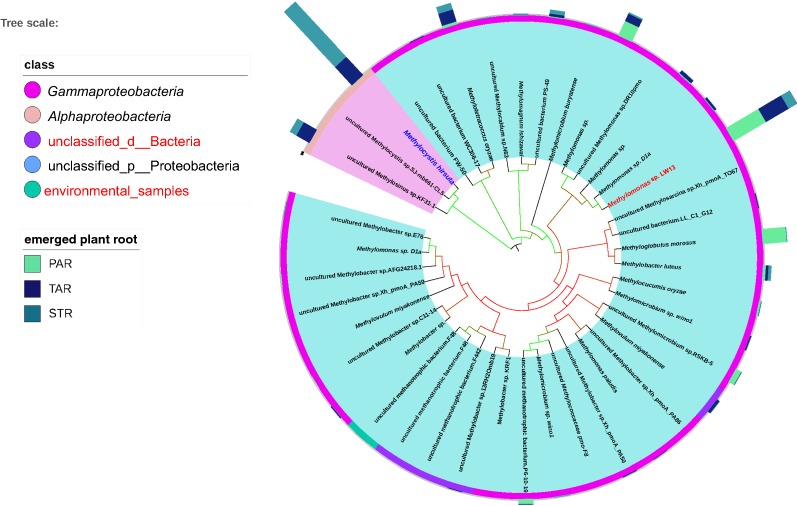
Neighbor-joining tree of methanotrophic phylotypes detected in the emergent plants based on the cDNA of *pmo*A genes. The blue part of the inner circle represents type I; the pink part represents type II. The different colors in the outer circle represent the corresponding class of OTU. Multi value bar charts represent the relative abundance of OTU in different plants

**Fig. 5:**
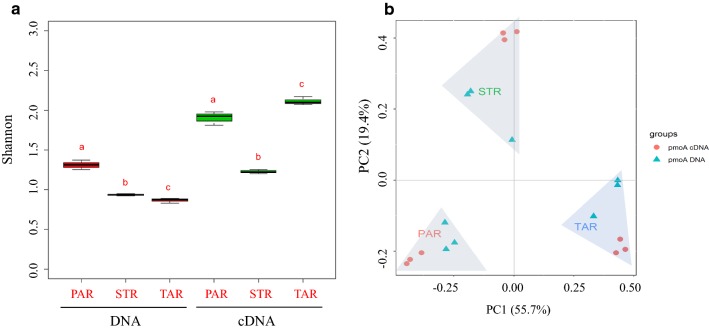
Alpha diversity and beta-diversity of aerobic methanotroph calculation based on *pmo*A. The OTUs evenness of root microbiotas is shown by Shannon(a). The red boxplot based on DNA; the green boxplot based on cDNA. Error bars represent the standard error of the mean. Bars with the different letter (a, b or c) within a panel (DNA or cDNA) are significantly different (p < 0.05). The distance between samples of principal coordinates analysis (PCoA) (b) calculated using Bray–Curtis index

By studying the community of methanotrophs at the transcriptional level, we can reflect more directly on the role of functional flora in controlling methane emissions. The total sequences, obtained by high throughput sequencing of *pmo*A on root cDNA, were classified into 41 OTUs (Fig. 4; Additional file 1: Table S2) assigned to 14 genera (Fig. 2b) and 5 classes (Fig. 4). The genus *Methylomonas* and *Methylococcaceae* which were type I methanotrophs in the *Gammaproteobacteria* and the genus *Methylocystis* and *Methylosinus* which were type II methanotrophs in the *Alphaproteobacteria* accounted for more than 80% of all samples.

The community structure varied greatly in different plants root (Fig. 2b). For *P. australis*, type I methanotrophs, including three main OTUs of *Methylomonas* sp. LW13 (Lontoh et al. 2000), *Methyloglobulus morosus* (Lontoh et al. 2000) and *Methylomonas* sp. (QBB78506.1), represented 75.1%; however, in *S. triqueter*, the most OTU of *Methylocystis hirsute* (Lontoh et al. 2000) affiliated with type II accounted for 68.0%. The proportions of type I and type II methanotrophs were almost equal in *T. angustifolia*. Two main OTUs, *Methylomonas* of Type I and *Methylocystis* of type II, accounted for 24.6% and 23.1%, respectively.

## Discussion

Research has shown that type I methanotrophs are always found in soils with a limited CH_4_ supply because they grow better than type II methanotrophs in a low-CH_4_ environment (Hanson and Hanson 1996). On the other hand, type II methanotrophs such as *Methylocystis* are usually found in high-CH_4_ systems (Shiau et al. 2018a, b). Recently, Kits et al. (2015) have used complete genome analysis to show that the type I methanotrophs *Methylomonas denitrificans* FJG1 possess related denitrification genes and demonstrate denitrification activity under hypoxic conditions. In addition, *Methylocystis* and *Methylosinus* of type II methanotrophs were found to be the predominant root-associated methanotrophs in rice paddy field (Bao et al. 2014b; Eller and Frenzel 2001; Qiu et al. 2009; Shinoda et al. 2019) and were identified as diazotrophic methanotrophs in rice root (Bao et al. 2014b; Shinoda et al. 2019). These results support that members of type I and type II methanotrophs inhabiting in aquatic plants in wetland. This study showed that type I methanotrophs dominate the root systems of the three species of emergent plants, which could easily be explained by the fact that the three species lived in the same water area and their aerenchyma was conducive to the proliferation of CH_4_, resulting in a low concentration of CH_4_ in this water area. However, when analyzing the active methanotrophs in cDNA, we found that the structure was significantly different from the DNA level, especially *S. triqueter*, whose active communities were mainly *Methylocystis* of type II methanotrophs. This leads us to the question: what factors affect the transcriptional activity of different methanotrophs? *Methylomonas* has been shown to be active in methane oxidation in the environments that are more neutral to alkaline in pH, such as a cave sys-tem, soda lake, and landfill cover soil (Cebron et al. 2007; Hutchens et al. 2004; Lin et al. 2004). On the other hand, Chen et al. (2008) determined that *Methylocystis* populations were predominant in the active methanotrophs in a range of peatlands in the United Kingdom. These results showed that environmental factors (e.g., pH) affect the activity of different groups of methanotrophs. The pH of sediments in WLSH was 8.0 (Additional file 1: Table S1), and all three plants in this study grew in the same slightly alkaline environment which is suitable for the habitation of *Methylomonas* (Liu et al. 2020). Nevertheless, the main active groups of these plant roots varied with different plant species in this research, which may be due to the transcriptional activity of methanotrophs being more sensitive to the change of microenvironment (root secretion, etc.) of the plant root. Rhizodermis cells secrete a wide range of compounds, including organic acid ions, inorganic ions, phytosiderophores, sugars, vitamins, amino acids, purines, and nucleosides, and the root cap produces polysaccharide mucilage (Dakora and Phillips 2002). These exudates have a great influence on root microhabitats. The production of organic acids, for example, may alter the pH of plant roots. Though the root exudates of emergent plants might get diluted in the wetland environment, the ability of the microbial capacity to adhere to the root of different plants may vary (Pietrangelo et al. 2018). Root secretion of *P. australis* may have little effect on the pH of the environment, but to *S. triqueter* and *T. angustifolia*, it could change the pH of the root surface, thereby affecting the community of active methanotrophs. CH_4_ emission may be one of the factors affecting the transcriptional activity of aerobic methanotrophs. As root-associated methanotrophs varies with plant species, it may affect the fluxes of plant mediated methane. Maltais-Landry et al. (2009) reported higher CH_4_ emissions from constructed wetlands planted with *P. australis* and *P. arundinacea* than for constructed wetlands planted with *T. angustifolia*. Therefore, the community structure of active aerobic methanotrophs is more valuable for the study of natural wetland CH_4_ emission.

As the microhabitat of bacterial life, the roots of emergent plants can be affected by various factors, such as the physical and chemical properties of sediments (Chen et al. 2018; Cui et al. 2017; Shiau et al. 2018a, b), vegetation types (Chen et al. 2019; Yoshida et al. 2014), growth period, and the change of microenvironment is bound to lead the change of root-associated bacterial community. Bulgarelli et al. (2013) found that the microbial community in different plant roots had its distinctive phylogenetic structure. Is the aerobic methanotrophs affected by plant host? Studies on rhizosphere sediments have shown that vegetation types affected the community structure of methanotrophs. Zhang et al. (2018) reported that plant species had a profound impact on methanotrophic communities, and each plant species in constructed wetlands contained a specific group of methanotrophs. Yoshida et al. (2014) studied the communities of methanotrophs in the leaves, submerged part and emerged part of different aquatic plants, and drew the same conclusion. Little work was done on root-associated methanotrophs in natural wetland. In this study, beta-diversity based on both *pmo*A DNA and cDNA (Fig. 5b) showed that the aerobic methanotrophic bacteria of three plants root vary with plant species. The root exudates of emergent plants in the wetland spread with water to other parts of the plant and surrounding sediments, which may be one reason why methanotrophic communities were different in rhizosphere and emerged part. In addition, aerobic methanotrophs form an important bridge between the global carbon and nitrogen cycles including denitrification and nitrogen fixation (Bao et al. 2014b; Stein and Klotz 2011). Further investigation should be conducted, such as transcriptomic and/or metaproteomic analysis of root-associated methanotrophs to clarify whether aerobic methanotrophs play a critical role to methane oxidation and denitrification or nitrogen fixation in natural wetland.

In this study, we demonstrated that the root-microhabitats of wetland emergent plants have significant influence on both the community structure and the active structure of aerobic methanotrophs. *Methylomonas* of type I methanotrophs was predominant in the root of three plant species in DNA level analysis. However, active root-associated *Methylocystis* of type II methanotrophs was predominant in the root of *S. triqueter*, as well as in *T. angustifolia*. The diversity and composition of active methanotrophs were dependent on plants species. The community of active methanotrophs were affected by the host plant. These results will lay the foundation for further studies on plant-mediated methane emissions from natural wetlands.

## Supplementary information

**Additional file 1: Table S1.** physicochemical characteristics of sediments and three plants roots in Wuliangsuhai wetland. **Table S2.** The most closely related *pmo*A sequences of OUTs in phylogenetic tree. **Fig. S1.** Alpha diversity of aerobic methanotroph calculation based on *pmo*A gene. The OTUs richness and OTUs evenness of root microbiotas is shown by chao (a) and Simpson index (b). The red boxplot based on DNA; the green boxplot based on cDNA. Error bars represent the standard error of the mean. Asterisks denote statistically significant differences between samples (**p < 0.01, *p < 0.05). Bars with the different letter (a, b or c) within a panel are significantly different between groups (p < 0.05). **Fig. S2.** Comparison of differences between groups is shown by ANOSIM. Distance calculated on OUT level of each sample groups.

## Data Availability

We declared that materials described in the manuscript, including all relevant raw data, will be freely available to any scientist wishing to use them for non-commercial purposes, without breaching participant confidentiality.
