# Supplementary material for: Diversity of active root-associated methanotrophs of three emergent plants in a eutrophic wetland in northern China
Source: AMB Express. 2020 Mar 14;10:48. doi: 10.1186/s13568-020-00984-x (PMC7070141; doi:10.1186/s13568-020-00984-x)
Supplement: Supplementary file 1 — Additional file 1: Table S1. physicochemical characteristics of sediments and three plants roots in Wuliangsuhai wetland. Table S2. The most closely related pmoA sequences of OUTs in phylogenetic tree. Fig. S1. Alpha diversity of aerobic methanotroph calculation based on pmoA gene. The OTUs richness and OTUs evenness of root microbiotas is shown by chao (a) and Simpson index (b). The red boxplot based on DNA; the green boxplot based on cDNA. Error bars represent the standard error of the mean. Asterisks denote statistically significant differences between samples (**p < 0.01, *p < 0.05). Bars with the different letter (a, b or c) within a panel are significantly different between groups (p < 0.05). Fig. S2. Comparison of differences between groups is shown by ANOSIM. Distance calculated on OUT level of each sample groups. [file 13568_2020_984_MOESM1_ESM.pptx]

## Slide 1
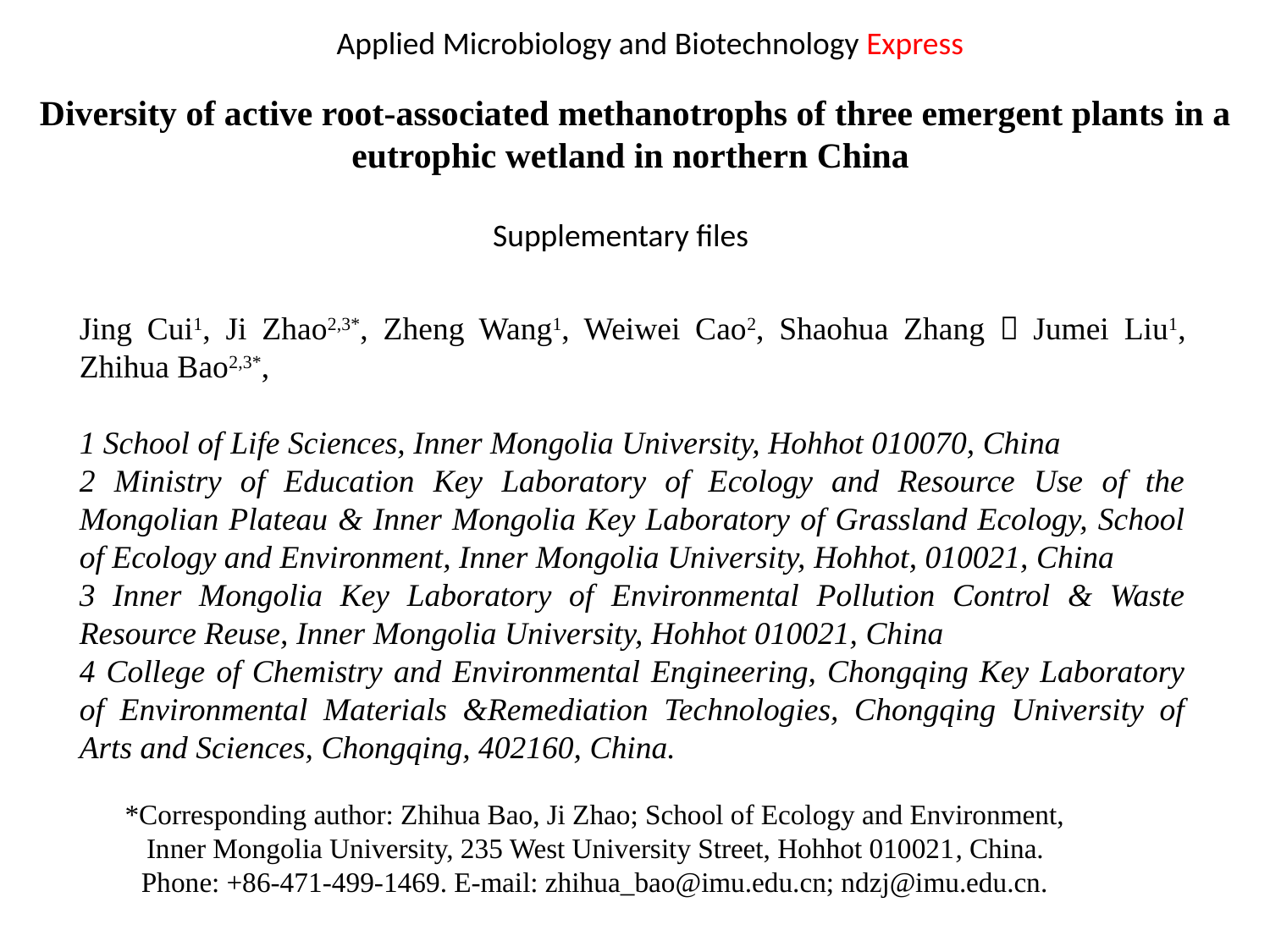

Applied Microbiology and Biotechnology Express
Diversity of active root-associated methanotrophs of three emergent plants in a eutrophic wetland in northern China
Supplementary files
Jing Cui1, Ji Zhao2,3*, Zheng Wang1, Weiwei Cao2, Shaohua Zhang，Jumei Liu1, Zhihua Bao2,3*,
1 School of Life Sciences, Inner Mongolia University, Hohhot 010070, China
2 Ministry of Education Key Laboratory of Ecology and Resource Use of the Mongolian Plateau & Inner Mongolia Key Laboratory of Grassland Ecology, School of Ecology and Environment, Inner Mongolia University, Hohhot, 010021, China
3 Inner Mongolia Key Laboratory of Environmental Pollution Control & Waste Resource Reuse, Inner Mongolia University, Hohhot 010021, China
4 College of Chemistry and Environmental Engineering, Chongqing Key Laboratory of Environmental Materials &Remediation Technologies, Chongqing University of Arts and Sciences, Chongqing, 402160, China.
*Corresponding author: Zhihua Bao, Ji Zhao; School of Ecology and Environment, Inner Mongolia University, 235 West University Street, Hohhot 010021, China. Phone: +86-471-499-1469. E-mail: zhihua_bao@imu.edu.cn; ndzj@imu.edu.cn.

## Slide 2
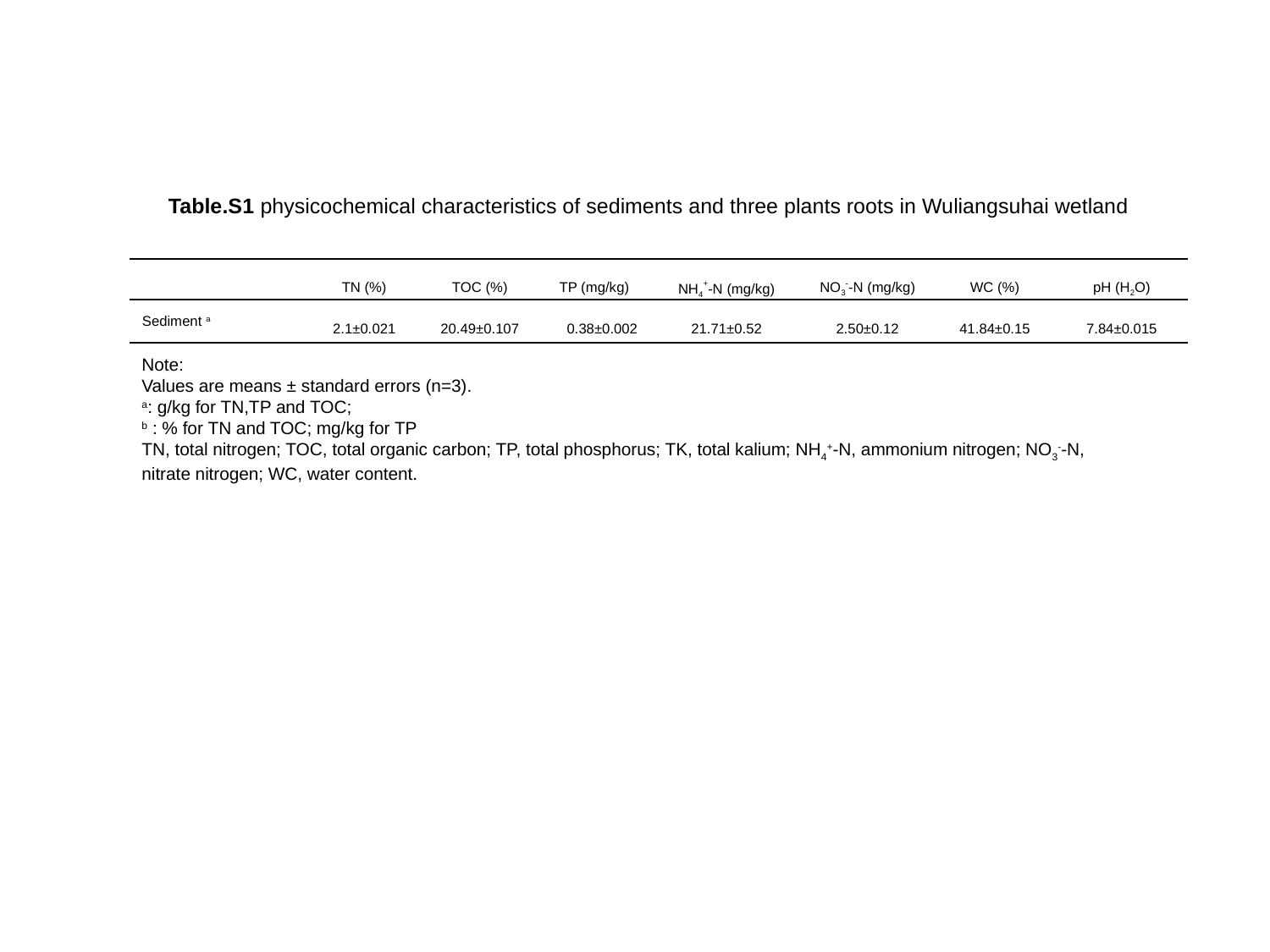

Table.S1 physicochemical characteristics of sediments and three plants roots in Wuliangsuhai wetland
| | TN (%) | TOC (%) | TP (mg/kg) | NH4+-N (mg/kg) | NO3--N (mg/kg) | WC (%) | pH (H2O) |
| --- | --- | --- | --- | --- | --- | --- | --- |
| Sediment a | 2.1±0.021 | 20.49±0.107 | 0.38±0.002 | 21.71±0.52 | 2.50±0.12 | 41.84±0.15 | 7.84±0.015 |
Note:
Values are means ± standard errors (n=3).
a: g/kg for TN,TP and TOC;
b : % for TN and TOC; mg/kg for TP
TN, total nitrogen; TOC, total organic carbon; TP, total phosphorus; TK, total kalium; NH4+-N, ammonium nitrogen; NO3--N, nitrate nitrogen; WC, water content.

## Slide 3
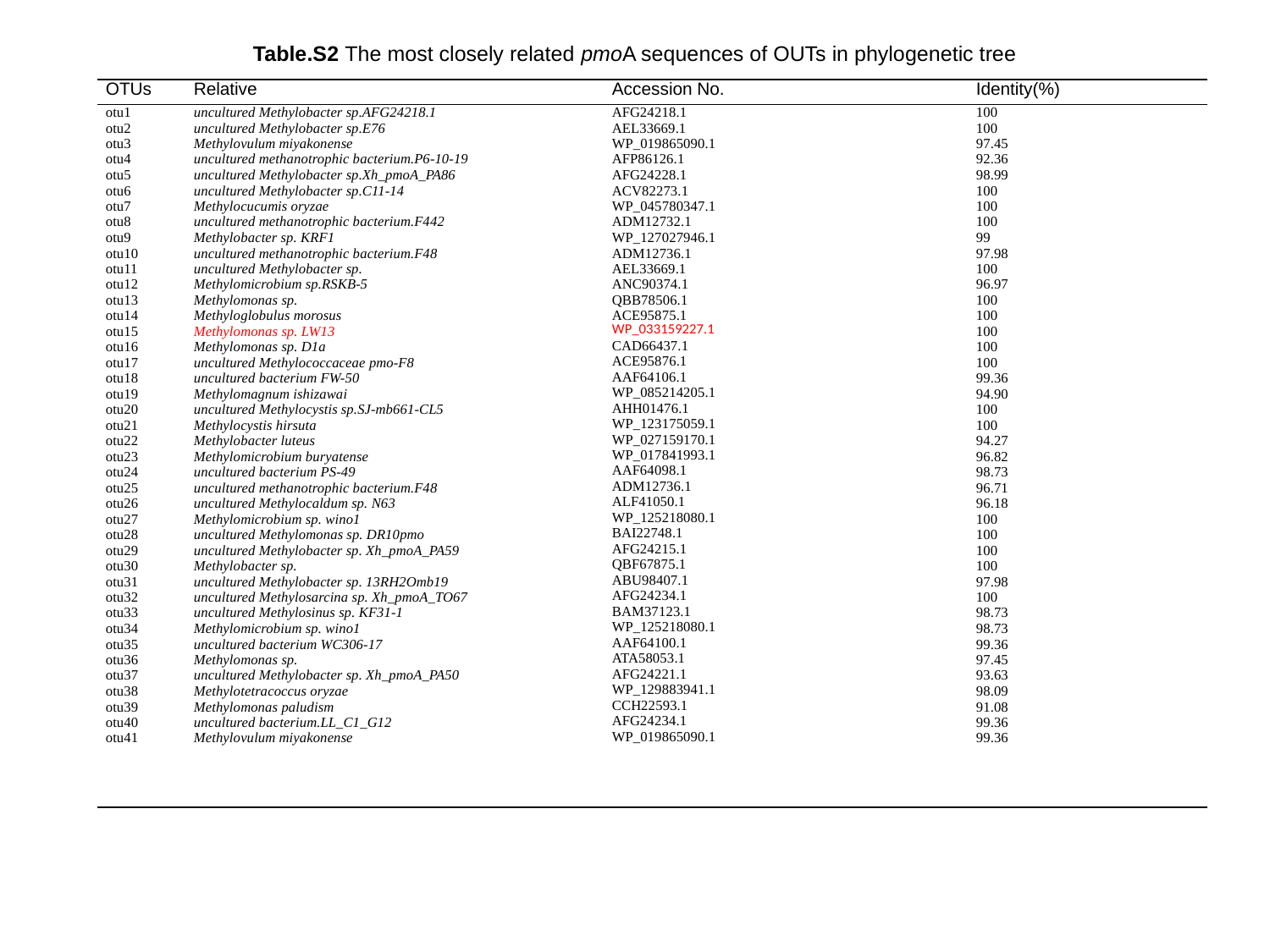

Table.S2 The most closely related pmoA sequences of OUTs in phylogenetic tree
| OTUs | Relative | Accession No. | Identity(%) |
| --- | --- | --- | --- |
| otu1 otu2 otu3 otu4 otu5 otu6 otu7 otu8 otu9 otu10 otu11 otu12 otu13 otu14 otu15 otu16 otu17 otu18 otu19 otu20 otu21 otu22 otu23 otu24 otu25 otu26 otu27 otu28 otu29 otu30 otu31 otu32 otu33 otu34 otu35 otu36 otu37 otu38 otu39 otu40 otu41 | uncultured Methylobacter sp.AFG24218.1 uncultured Methylobacter sp.E76 Methylovulum miyakonense uncultured methanotrophic bacterium.P6-10-19 uncultured Methylobacter sp.Xh\_pmoA\_PA86 uncultured Methylobacter sp.C11-14 Methylocucumis oryzae uncultured methanotrophic bacterium.F442 Methylobacter sp. KRF1 uncultured methanotrophic bacterium.F48 uncultured Methylobacter sp. Methylomicrobium sp.RSKB-5 Methylomonas sp. Methyloglobulus morosus Methylomonas sp. LW13 Methylomonas sp. D1a uncultured Methylococcaceae pmo-F8 uncultured bacterium FW-50 Methylomagnum ishizawai uncultured Methylocystis sp.SJ-mb661-CL5 Methylocystis hirsuta Methylobacter luteus Methylomicrobium buryatense uncultured bacterium PS-49 uncultured methanotrophic bacterium.F48 uncultured Methylocaldum sp. N63 Methylomicrobium sp. wino1 uncultured Methylomonas sp. DR10pmo uncultured Methylobacter sp. Xh\_pmoA\_PA59 Methylobacter sp. uncultured Methylobacter sp. 13RH2Omb19 uncultured Methylosarcina sp. Xh\_pmoA\_TO67 uncultured Methylosinus sp. KF31-1 Methylomicrobium sp. wino1 uncultured bacterium WC306-17 Methylomonas sp. uncultured Methylobacter sp. Xh\_pmoA\_PA50 Methylotetracoccus oryzae Methylomonas paludism uncultured bacterium.LL\_C1\_G12 Methylovulum miyakonense | AFG24218.1 AEL33669.1 WP\_019865090.1 AFP86126.1 AFG24228.1 ACV82273.1 WP\_045780347.1 ADM12732.1 WP\_127027946.1 ADM12736.1 AEL33669.1 ANC90374.1 QBB78506.1 ACE95875.1 WP\_033159227.1 CAD66437.1 ACE95876.1 AAF64106.1 WP\_085214205.1 AHH01476.1 WP\_123175059.1 WP\_027159170.1 WP\_017841993.1 AAF64098.1 ADM12736.1 ALF41050.1 WP\_125218080.1 BAI22748.1 AFG24215.1 QBF67875.1 ABU98407.1 AFG24234.1 BAM37123.1 WP\_125218080.1 AAF64100.1 ATA58053.1 AFG24221.1 WP\_129883941.1 CCH22593.1 AFG24234.1 WP\_019865090.1 | 100 100 97.45 92.36 98.99 100 100 100 99 97.98 100 96.97 100 100 100 100 100 99.36 94.90 100 100 94.27 96.82 98.73 96.71 96.18 100 100 100 100 97.98 100 98.73 98.73 99.36 97.45 93.63 98.09 91.08 99.36 99.36 |

## Slide 4
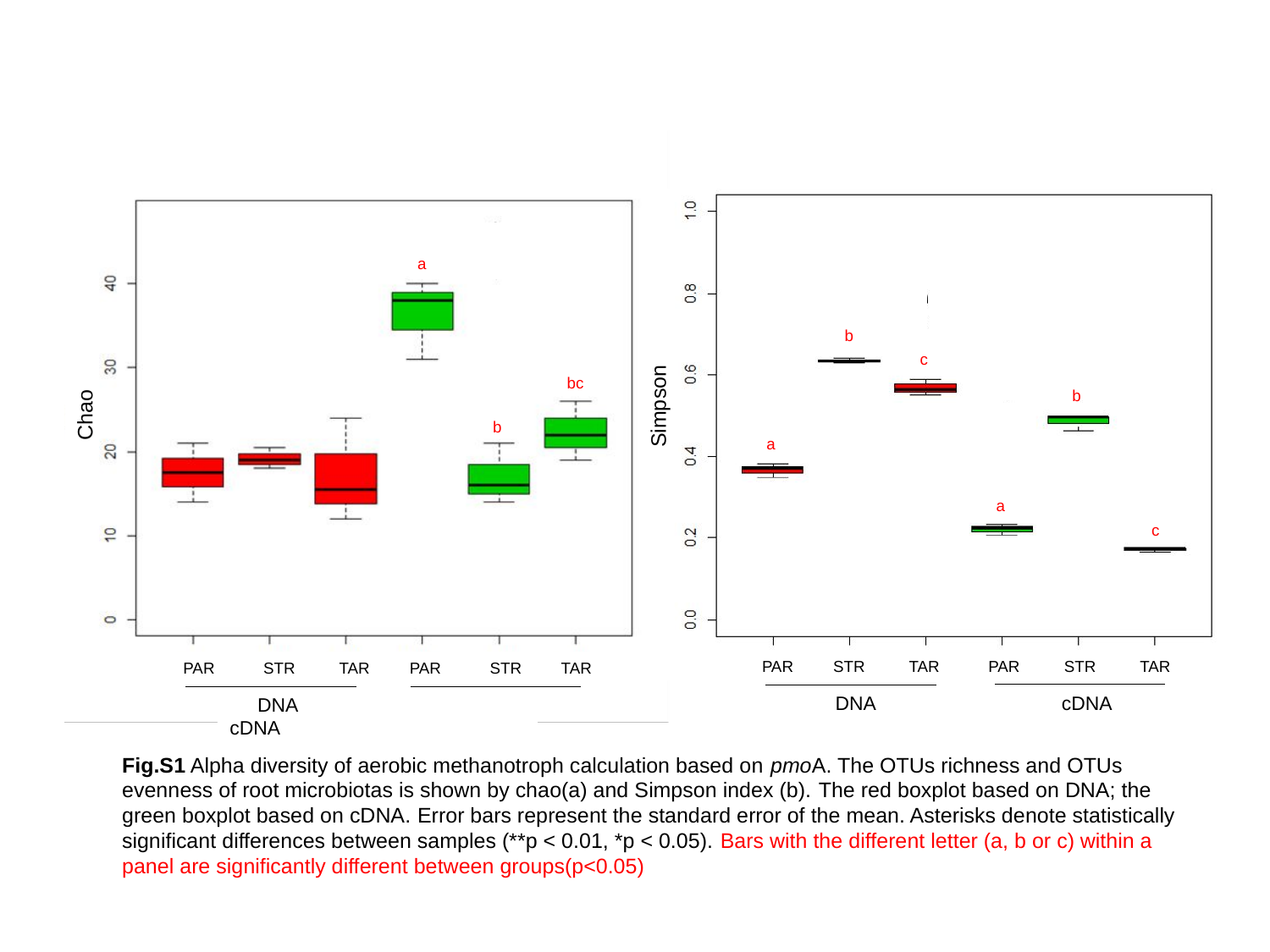

PAR STR TAR PAR STR TAR
 DNA cDNA
Chao
PAR STR TAR PAR STR TAR
Simpson
a
b
c
bc
b
b
a
a
c
DNA cDNA
Fig.S1 Alpha diversity of aerobic methanotroph calculation based on pmoA. The OTUs richness and OTUs evenness of root microbiotas is shown by chao(a) and Simpson index (b). The red boxplot based on DNA; the green boxplot based on cDNA. Error bars represent the standard error of the mean. Asterisks denote statistically significant differences between samples (**p < 0.01, *p < 0.05). Bars with the different letter (a, b or c) within a panel are significantly different between groups(p<0.05)

## Slide 5
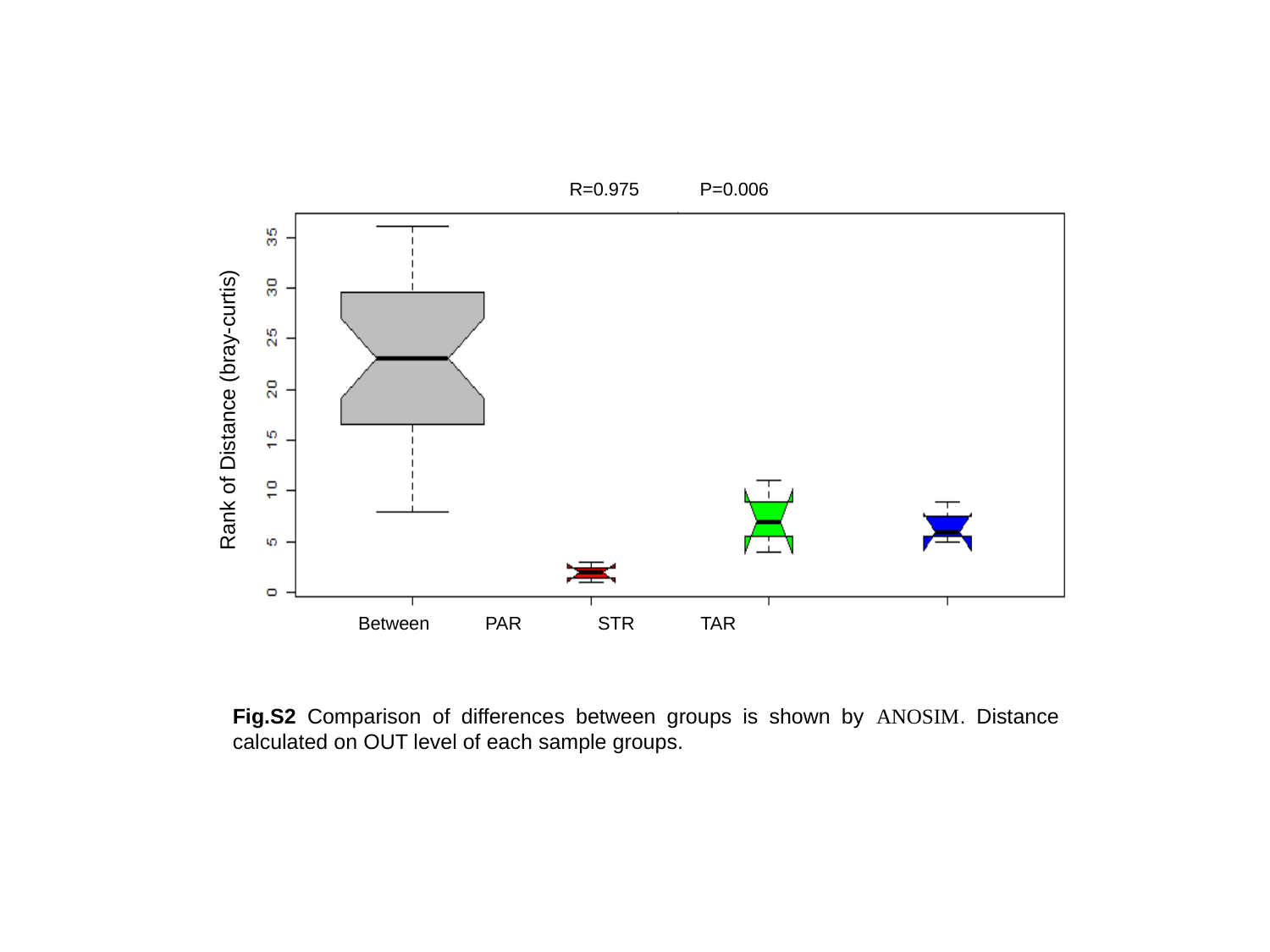

R=0.975 P=0.006
Rank of Distance (bray-curtis)
Between PAR STR TAR
Fig.S2 Comparison of differences between groups is shown by ANOSIM. Distance calculated on OUT level of each sample groups.
